# MambaPhase: deep learning for liquid–liquid phase separation protein classification

**DOI:** 10.1093/bib/bbaf230

**Published:** 2025-05-27

**Authors:** Jianwei Huang, Youli Zhang, Shulin Ren, Ziyang Wang, Xiaocheng Jin, Xiaoli Lu, Yu Zhang, Xiaoping Min, Shengxiang Ge, Jun Zhang, Ningshao Xia

**Affiliations:** Institute of Artificial Intelligence, School of Informatics, Xiamen University, No. 422 Siming South Rd, 361005, Xiamen, Fujian, China; National Institute of Diagnostics and Vaccine Development in Infectious Diseases, School of Public Health, Xiamen University, No. 422 Siming South Rd, 361005, Xiamen, Fujian, China; State Key Laboratory of Vaccines for Infectious Diseases, Xiang An Biomedicine Laboratory, School of Public Health, Xiamen University, No. 422 Siming South Rd, 361005, Xiamen, Fujian, China; National Institute of Diagnostics and Vaccine Development in Infectious Diseases, School of Public Health, Xiamen University, No. 422 Siming South Rd, 361005, Xiamen, Fujian, China; State Key Laboratory of Vaccines for Infectious Diseases, Xiang An Biomedicine Laboratory, School of Public Health, Xiamen University, No. 422 Siming South Rd, 361005, Xiamen, Fujian, China; State Key Laboratory of Vaccines for Infectious Diseases, Xiang An Biomedicine Laboratory, School of Public Health, Xiamen University, No. 422 Siming South Rd, 361005, Xiamen, Fujian, China; Information and Networking Center, Xiamen University, No. 422 Siming South Rd, 361005, Xiamen, Fujian, China; Institute of Artificial Intelligence, School of Informatics, Xiamen University, No. 422 Siming South Rd, 361005, Xiamen, Fujian, China; National Institute of Diagnostics and Vaccine Development in Infectious Diseases, School of Public Health, Xiamen University, No. 422 Siming South Rd, 361005, Xiamen, Fujian, China; State Key Laboratory of Vaccines for Infectious Diseases, Xiang An Biomedicine Laboratory, School of Public Health, Xiamen University, No. 422 Siming South Rd, 361005, Xiamen, Fujian, China; National Institute of Diagnostics and Vaccine Development in Infectious Diseases, School of Public Health, Xiamen University, No. 422 Siming South Rd, 361005, Xiamen, Fujian, China; State Key Laboratory of Vaccines for Infectious Diseases, Xiang An Biomedicine Laboratory, School of Public Health, Xiamen University, No. 422 Siming South Rd, 361005, Xiamen, Fujian, China; Information and Networking Center, Xiamen University, No. 422 Siming South Rd, 361005, Xiamen, Fujian, China; Institute of Artificial Intelligence, School of Informatics, Xiamen University, No. 422 Siming South Rd, 361005, Xiamen, Fujian, China; National Institute of Diagnostics and Vaccine Development in Infectious Diseases, School of Public Health, Xiamen University, No. 422 Siming South Rd, 361005, Xiamen, Fujian, China; State Key Laboratory of Vaccines for Infectious Diseases, Xiang An Biomedicine Laboratory, School of Public Health, Xiamen University, No. 422 Siming South Rd, 361005, Xiamen, Fujian, China; Institute of Artificial Intelligence, School of Informatics, Xiamen University, No. 422 Siming South Rd, 361005, Xiamen, Fujian, China; National Institute of Diagnostics and Vaccine Development in Infectious Diseases, School of Public Health, Xiamen University, No. 422 Siming South Rd, 361005, Xiamen, Fujian, China; State Key Laboratory of Vaccines for Infectious Diseases, Xiang An Biomedicine Laboratory, School of Public Health, Xiamen University, No. 422 Siming South Rd, 361005, Xiamen, Fujian, China; National Institute of Diagnostics and Vaccine Development in Infectious Diseases, School of Public Health, Xiamen University, No. 422 Siming South Rd, 361005, Xiamen, Fujian, China; State Key Laboratory of Vaccines for Infectious Diseases, Xiang An Biomedicine Laboratory, School of Public Health, Xiamen University, No. 422 Siming South Rd, 361005, Xiamen, Fujian, China; National Institute of Diagnostics and Vaccine Development in Infectious Diseases, School of Public Health, Xiamen University, No. 422 Siming South Rd, 361005, Xiamen, Fujian, China; State Key Laboratory of Vaccines for Infectious Diseases, Xiang An Biomedicine Laboratory, School of Public Health, Xiamen University, No. 422 Siming South Rd, 361005, Xiamen, Fujian, China; National Institute of Diagnostics and Vaccine Development in Infectious Diseases, School of Public Health, Xiamen University, No. 422 Siming South Rd, 361005, Xiamen, Fujian, China; State Key Laboratory of Vaccines for Infectious Diseases, Xiang An Biomedicine Laboratory, School of Public Health, Xiamen University, No. 422 Siming South Rd, 361005, Xiamen, Fujian, China

**Keywords:** liquid–liquid phase separation (LLPS), machine learning, protein phase separation, drug delivery

## Abstract

Liquid–liquid phase separation plays a critical role in cellular processes, including protein aggregation and RNA metabolism, by forming membraneless subcellular structures. Accurate identification of phase-separated proteins is essential for understanding and controlling these processes. Traditional identification methods are effective but often costly and time-consuming. The recent machine learning methods have reduced these costs, but most models are restricted to classifying scaffold and client proteins with limited experimental conditions. To address this limitation, we developed a Mamba-based encoder using contrastive learning that incorporates separation probability, protein type, and experimental conditions. **Our model achieved 95.2% accuracy in predicting phase-separated proteins** and an ROCAUC score of 0.87 in classifying scaffold and client proteins. Further validation in the DgHBP-2 drug delivery system demonstrated its potential for condition modulation in drug development. This study provides an effective framework for the accurate identification and control of phase separation, facilitating advancements in biomedical research and therapeutic applications.

## Introduction

Liquid–liquid phase separation (LLPS) is a mechanism by which subcellular structures such as liquid droplets, nucleoli, and P granules form spontaneously within cells without membrane boundaries [1]. It involves specific molecules separating from each other within a liquid medium due to intermolecular interactions, thus creating distinct liquid-phase regions. This phenomenon resembles the separation of oil droplets from water when mixed [2]. Scaffold proteins and client proteins are two key components in this process. Scaffold proteins provide structural support and frameworks for droplet formation, and recruit client proteins into droplets, thereby affecting droplet formation and stability [3]. Client proteins, on the other hand, are recruited into liquid-phase condensates through specific interactions with scaffold proteins, modulating condensate properties such as viscoelasticity or surface tension, or participating in particular biochemical reactions, thus regulating condensate function. The synergy between scaffold and client proteins enhances the efficiency and specificity of pharmaceutical carrier systems, whose targeted delivery capabilities strongly depend on precise LLPS control [4].

LLPS closely relates to intermolecular interactions and protein sequence composition. Traditional biological methods for detecting LLPS rely mainly on fluorescence-based observation of protein aggregation patterns. However, these experimental methods are costly and time-consuming [5]. Computational approaches have recently emerged to overcome experimental limitations, supporting rapid identification and screening of LLPS-prone proteins. Early-stage LLPS prediction methods, including PLAAC, PScore, and catGRANULE, primarily utilized physicochemical properties—such as hydrophobicity, charge, and content of intrinsically disordered regions (IDRs)—for sequence analysis and prediction. These tools initially improved the efficiency of LLPS identification and laid a crucial foundation for subsequent methodological advances [6–8]. Nevertheless, due to the limited availability of data in early public LLPS databases, predictive models built solely on physicochemical features generally showed insufficient accuracy in practical applications.

In recent years, the continuous expansion and development of LLPS-related databases, such as DrLLPS, LLPSDB, and LLPSDB2.0 [9, 10], have provided abundant experimental data supporting a new generation of predictive models. Advanced algorithms, including Opt_PredLLPS, PredLLPS_PSSM, and PSPredictor, increasingly integrate richer protein sequence datasets, evolutionary information, multilevel feature extraction, sophisticated embedding approaches, and deep learning architectures (e.g. convolutional neural networks, Bidirectional Long Short-Term Memory) to significantly enhance prediction model generalizability [11, 12]. Among these new algorithms, Opt_PredLLPS not only classifies proteins based on LLPS potential, but also predicts whether specific partner interactions are required for proteins to undergo phase separation. PSPredictor combines sequence composition and evolutionary conservation information, substantially improving accuracy and robustness in recognizing LLPS proteins. In addition, other recently proposed tools such as MolPhase, FLFB, and PSPHunter have focused on improving LLPS prediction accuracy and expanding the functional insights provided by these models. Specifically, MolPhase integrates comprehensive biochemical and biophysical features, combining experimental validations with predictions to accurately identify proteins prone to LLPS and dissect protein–protein interaction modes driving phase separation [13]. Furthermore, FLFB systematically distinguishes LLPS-prone proteins from amyloid fibril-forming proteins using carefully selected sequence-derived features, thus addressing the critical need to differentiate distinct aggregation pathways to understand their biological functions and pathological mechanisms [14]. Finally, PSPHunter leverages advanced machine-learning techniques to precisely identify key amino acid residues crucial for LLPS, which has been experimentally verified to modulate cellular phenotypes, transcription regulation, and disease progression when these residues are mutated [15]. Collectively, these recent methods enhance prediction accuracy, broaden the application contexts of LLPS models, and provide more refined molecular insights into phase separation behavior, advancing our understanding of the determinants and functional consequences of biomolecular LLPS. Despite these advances, tools currently available lack systematic modeling of crucial environmental parameters (e.g. pH and salt concentration) explicitly for LLPS processes [16]. Many existing models classify only certain proteins without incorporating background information of LLPS conditions, thus providing limited guidance in practical applications. Indeed, precise control of environmental conditions such as pH and salt concentration is crucial for accurately regulating LLPS, particularly for finely controlled drug-delivery applications [17]. By adjusting environmental parameters and comprehensively analyzing scaffold-client protein interactions, researchers can precisely control LLPS processes, enabling efficient design and optimization of drug delivery systems [18].

Recently, large language models, such as Evolutionarily Scaled Model (ESM) and ProteinBERT, have shown remarkable advantages when working with small datasets, benefiting from extensive prior knowledge embedded in their pretrained representations. Several studies have already explored their potential applications in LLPS protein prediction [19, 20]. Research suggests large language models demonstrate significant strength in tasks involving LLPS protein sequence classification [21]. Therefore, in this study, we introduce the language model ESM and fine-tune it using the DrLLPS database to extract features more efficiently and precisely for LLPS protein predictions. However, we still face significant challenges, such as limited data availability and severe imbalance between positive and negative samples in existing LLPS databases. To tackle these issues, we further introduce contrastive learning to construct a novel encoder more effectively capturing discriminative features among different categories of LLPS proteins [22]. To enhance the efficiency of encoder training and inference, we adopt the recently proposed Mamba algorithm. Mamba achieves similar performances to Transformer-based models while substantially reducing GPU resource consumption [23]. These methodological advances create an important foundation for further optimizing LLPS classifiers and developing an integrated LLPS prediction platform that simultaneously incorporates scaffold proteins, client proteins, and environmental parameters such as pH and salt concentration.

**Figure 1:**
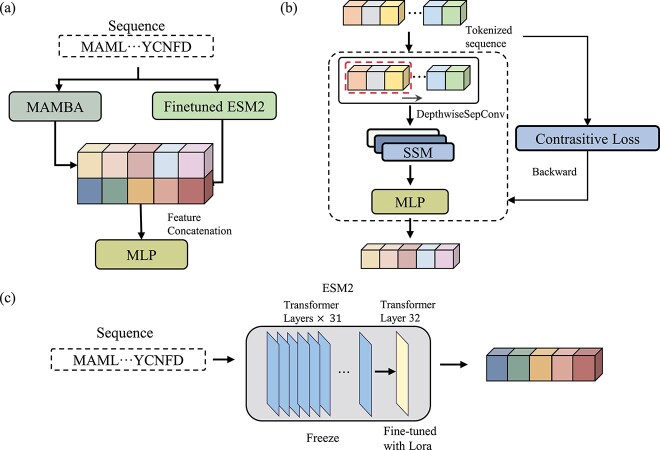
(a) The model encodes protein sequences separately using the MAMBA model and LoRA-tuned ESM2 model, fuses the extracted features, and performs final classification through an MLP. (b) The MAMBA model extracts local and dynamic sequence features via DepthwiseSepConv and a selective state space model. (c) Only the last transformer layer of ESM2 is fine-tuned using LoRA to improve efficiency and feature generalization.

Here, we proposed the “MambaPhase” model. This model combines the large language model ESM with the Mamba model to extract specific sequence features. And we integrated the DrLLPS and LLPSDB2.0 databases and divided the phase separation data into new datasets based on three ranges of salt concentrations and pH values. As shown in Fig. 1, our model can predict whether a protein is a phase separation protein and determine whether it is a scaffold or client protein while assessing the likelihood of phase separation at different salt concentrations and pH values [24, 25]. This functionality enables researchers to control the phase separation process by adjusting the salt concentration and pH value, facilitating the construction of more controllable pharmaceutical systems.

## Method

### MambaPhase

LLPS proteins are typically involved in complex intracellular biological processes [26]. Different types of LLPS proteins, such as scaffold proteins and client proteins, as well as proteins undergoing phase separation under different pH values and salt concentrations, exhibit differences in amino acid preferences and physicochemical properties in their sequences [27]. To effectively analyze these sequences, we developed a Mamba-based sequence feature encoder and designed a custom loss function, denoted as $\mathcal{L}$, to capture the characteristic differences between various LLPS proteins. The loss function is formulated as follows: 


(1) \begin{align*}& \mathcal{L} = -\frac{1}{N} \sum_{i=1}^{N} \frac{1}{|P(i)|} \sum_{p \in P(i)} \left(\text{sim}(z_{i}, z_{p}) - \log \sum_{a=1}^{N} e^{\text{sim}(z_{i}, z_{a})} \right),\end{align*}

where $N$ represents the total number of samples, $i$ indicates the current sample, and $P(i)$ is the set of samples belonging to the same category as sample $i$. The vectors $z_{i}$ and $z_{p}$ denote the feature representations of samples $i$ and $p$, respectively, with similarity $\text{sim}(z_{i}, z_{p})$ computed as their dot product. In this formulation, the first term enhances intraclass similarity by maximizing feature similarity between samples of the same category, while the second term normalizes similarities across all samples, thus promoting interclass separation.

Figure 1 delineates the architecture of MambaPhase, our proposed algorithmic model. Built upon the Mamba neural network—an innovative deep learning framework designed to address computational inefficiencies in processing long sequential data—the model introduces a *Selective State Space Model* (SSM). This mechanism enables dynamic filtration of crucial sequence elements while maintaining computational scalability through linear time complexity $\mathcal{O}(N)$, where $N$ now denotes sequence length [28]. Distinct from conventional Transformer architectures and other sequence analysis models relying on exhaustive pairwise computations, Mamba’s selective mechanism achieves superior computational efficiency. This allows our custom-crafted loss function (Equation 1) to precisely optimize feature differentiation between distinct LLPS proteins while preserving a global receptive field.

In the implementation, the model initially employs an embedding layer with a 64-dimensional feature representation to transform amino acids into continuous vectors. Using a vocabulary of 21 amino acid symbols (20 standard amino acids plus an unknown token), these sequences are subsequently processed through a depthwise separable one-dimensional convolutional operation with a kernel of size $3\times 1$ and 128 filters to extract local features [29]. The 1D convolution operation is defined as follows: 


(2) \begin{align*}& \text{Conv1d}(x) = \text{DepthwiseSepConv}(x; W_{\text{k}}, W_{\text{p}}) + b,\end{align*}

where $\text{DepthwiseSepConv}$ consists of a depthwise convolution with kernel $W_{\text{k}} \in \mathbb{R}^{3 \times 1}$, capturing spatial information, followed by a pointwise convolution with filter $W_{\text{p}} \in \mathbb{R}^{d \times 128}$ (where $d$ is the number of input channels) to mix inter-channel features. The *groups* parameter is configured for depthwise convolution, convolving each input channel separately. A bias term $b$ is included for model flexibility.

To better model temporal dynamics of sequences, we introduce a selective version of the SSM within the Mamba module. Specifically, we employ eight state space parameters ($N_{\text{ssm}}=8$) and a rank-1 discretization projection decomposition ($r_{\text{dt}}=1$) to efficiently parameterize sequence dynamics. The parameter matrix $A$ and discretization step parameter $\delta $ are computed as follows: 


(3) \begin{align*} & A = -\exp(A_{\text{log}}), \end{align*}

(4) \begin{align*} & \delta = \text{softplus}(\text{dt}{\_}\text{proj}(\delta)). \end{align*}

In Equation 3, matrix $A$ is initialized based on the HiPPO-LegS formulation. In Equation 4, vector $\delta $ undergoes projection through $\text{dt}{\_}\text{proj}$ followed by a softplus transform. With these parameters, the selective SSM dynamically updates through the Selective Scan mechanism: 


(5) \begin{align*}& \text{SSM}(x) = \text{SelectiveScan}(x, \delta, A),\end{align*}

where the selective scanning mechanism utilizes parameters $A$ and $\delta $ to track dynamic features and predict LLPS characteristics.

The MambaPhase architecture consists of a six-layer backbone with dual residual blocks per layer, utilizing RMSNorm normalization ($\epsilon =1\times 10^{-5}$) for stable gradient propagation. The model expands embedding dimensions from 64 to 128 in feed-forward layers, culminating in 256-dimensional discriminative outputs. Training employs AdamW optimization ($\beta _{1}=0.9, \beta _{2}=0.98$), cosine-decayed learning rate scheduling ($3\times 10^{-4}$ initial, $1\times 10^{-5}$ final), batch size of 32, dropout probability of 0.3, weight decay of 0.1, and lasts 20 epochs. This yields an efficient 48-million parameter architecture while maintaining representational capacity for LLPS pattern recognition.

### ESM block

ESM is a deep learning model trained on large-scale protein sequence data, capable of capturing evolutionary information and complex structure–function relationships within protein sequences. We developed a fine-tuned model based on the ESM framework [30], which applies the rich knowledge from other protein domains to the prediction of LLPS proteins, thereby improving the prediction accuracy and enhancing the model’s generalization capability. Moreover, this approach can make full use of the extensive existing protein data even when data are limited, providing new perspectives and methods for LLPS research.

As a large-scale language model, fine-tuning the last layer of ESM2 requires sufficient data to influence parameter adjustment. However, direct full-scale fine-tuning consumes substantial computational resources. Without employing other techniques, this becomes unfeasible. Therefore, we introduced the low-rank adaptation (LoRA) technique [31], which reduces the number of updated model parameters through low-rank matrix decomposition, significantly lowering computational resource consumption. This approach effectively reduces training time and computational cost. Specifically, this study performs a low-rank approximation on the last layer of the model, defining the weight matrix $ W $ as 


(6) \begin{align*}& W = W_{0} + \Delta W,\end{align*}

In equation 6, $ W_{0} $ is the original weight matrix, and $ \Delta W $ is the weight change matrix obtained through low-rank decomposition, defined as 


(7) \begin{align*}& \Delta W = A B,\end{align*}

In equation 7, $ A $ and $ B $ are low-rank matrices, and $\text{rank}(A) = \text{rank}(B) = r \ll \min (m, n)$. In this way, parameter updates only involve these two low-rank matrices $ A $ and $ B $, significantly reducing computational complexity. We used this approach to fine-tune the last layer of the ESM model.

To improve the model’s understanding of LLPS protein patterns, we employed the Masked Language Model training method [32]. Specifically, we randomly mask parts of the amino acid sequences and then train the model to predict them. This method allows the model not only to capture the general patterns of LLPS proteins but also to understand their unique details and characteristics. We concatenated the features extracted from our fine-tuned model with those from the Mamba model to complete the feature fusion. Subsequently, we passed the concatenated features through a multilayer perceptron to generate the final output.

## Results

### Dataset construction

**Figure 2:**
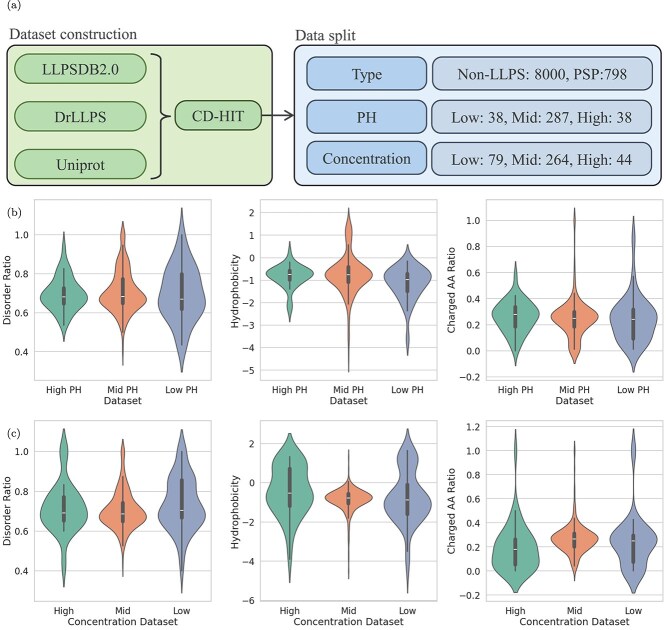
(a) The process of constructing our dataset, where CD-HIT v. 4.8.1 was used for clustering with parameters set to -c 0.4 -n 2; (b-c) Radar charts visualizing hydrophilicity, disordered regions, and charge content, demonstrating the validity of experimental condition classification under (b) different pH conditions and (c) different concentration conditions.

As illustrated in Fig. 2(a), the datasets used in this study were mainly derived from the LLPSDB 2.0 and DrLLPS databases. To ensure data validity and integrity, initial filtering was performed to remove protein sequences containing invalid amino acid characters or sequences with inappropriate lengths (shorter than 50 or longer than 5000 amino acids). Furthermore, to maintain dataset independence and prevent data leakage during model training, redundant sequences were identified and excluded using the CD-Hit algorithm with a sequence identity threshold of 40% [33]. After the above procedures, a benchmark dataset containing a total of 798 protein sequences capable of undergoing LLPS was established as the positive sample set. Protein sequences used as negative samples (proteins unlikely to undergo phase separation) were collected from the UniProt database. Initially, 22 000 protein sequences with experimentally resolved three-dimensional structures and single-domain annotations were extracted. These proteins were selected as negative samples because previous studies have shown that proteins associated with LLPS are typically enriched in IDRs, whereas proteins with fully experimentally resolved three-dimensional structures—especially single-domain proteins—usually lack significant disordered regions and are therefore less likely to undergo phase separation [34]. Subsequently, to further reduce sequence redundancy within the negative sample dataset, clustering and removal of redundant sequences were conducted using the CD-Hit software at a more rigorous sequence identity threshold of 30%, thus yielding a final negative dataset consisting of 8000 sequences. Additionally, scaffold and client protein datasets employed in this study were obtained from the seq2phase resource, containing 90 scaffold proteins and 2700 client proteins. This dataset was constructed by integrating protein sequences from SwissProt along with explicitly annotated scaffold and client proteins from the DrLLPS database. Redundant entries were likewise filtered using CD-Hit with a 40% sequence identity threshold.

To mitigate potential negative effects arising from class imbalance, a balanced dataset was generated using a 1:1 ratio between positive and negative samples. A 10-fold cross-validation strategy was applied for model assessment, and negative samples were selected through a randomized sampling approach. Specifically, for each cross-validation round, a distinct subset of negative samples was chosen based on different random seeds, thus ensuring both reliability and objectivity during model evaluation.

Subsequently, we constructed new datasets for pH and salt concentration classification. Specifically, we extracted protein data from the LLPSDB 2.0 database and divided it into distinct intervals to create separate datasets for pH and salt concentration conditions, which were then used to train corresponding classification models. Previous studies have shown that the LLPS behavior of proteins is influenced by a variety of physicochemical properties, particularly the proportion of IDRs (disorder ratio), hydrophobicity, and the ratio of charged amino acids (charged amino acid ratio). These properties affect the ability of proteins to form multivalent interactions, which in turn significantly impacts their tendency to undergo phase separation [35].

To ensure the accuracy and reliability of model training, we first employed the CD-HIT algorithm with a sequence identity threshold of 40% to remove redundant sequences and minimize bias caused by high sequence similarity. Following this preprocessing step, we conducted statistical and visual analyses of the aforementioned three key physicochemical properties on the deduplicated datasets. The results revealed that when pH values were divided into intervals of 5 and 7, and salt concentrations were categorized into 0.1 and 0.3 mM/L, significant differences in disorder ratio, hydrophobicity, and charged amino acid ratio were observed between the groups. This indicates that our partitioning strategy effectively captures biologically relevant variation in physicochemical properties, which can enhance the discriminative power of the classification models. Figure 2b and c illustrates the distribution of these properties under different experimental conditions, further validating the rationality of the selected grouping criteria. Ultimately, the pH dataset comprised 38 entries in the high-pH group, 287 in the medium group, and 38 in the low-pH group; the salt concentration dataset consisted of 79 entries in the high-salt group, 264 in the medium-salt group, and 44 in the low-salt group.

### Phase separation prediction

In our study, a model to identify and analyze the LLPS characteristics of proteins were developed. The primary objective of the model is to identify proteins with phase separation potential and distinguish them as either client proteins or scaffold proteins. After initial identification, we assessed the likelihood of these proteins undergoing phase separation under different pH and salt concentration conditions. To comprehensively evaluate our model performance, we employed multiple metrics, including Accuracy (Acc), Precision, F1-Score, and Matthews correlation coefficient (MCC), which are defined as follows: 


(8) \begin{align*} & \text{Accuracy} = \frac{TP + TN}{TP + TN + FP + FN}, \end{align*}

(9) \begin{align*} & \text{Precision} = \frac{TP}{TP + FP}, \end{align*}

(10) \begin{align*} & \text{Recall} = \frac{TP}{TP + FN}, \end{align*}

(11) \begin{align*} & \text{F1-Score} = 2 \times \frac{\text{Precision} \times \text{Recall}}{\text{Precision} + \text{Recall}}, \end{align*}

(12) \begin{align*} & \text{MCC} = \frac{TP \times TN - FP \times FN}{\sqrt{(TP+FP)(TP+FN)(TN+FP)(TN+FN)}}, \end{align*}

where $ TP $, $ TN $, $ FP $, and $ FN $ denote the numbers of true positives, true negatives, false positives, and false negatives, respectively.

**Figure 3:**
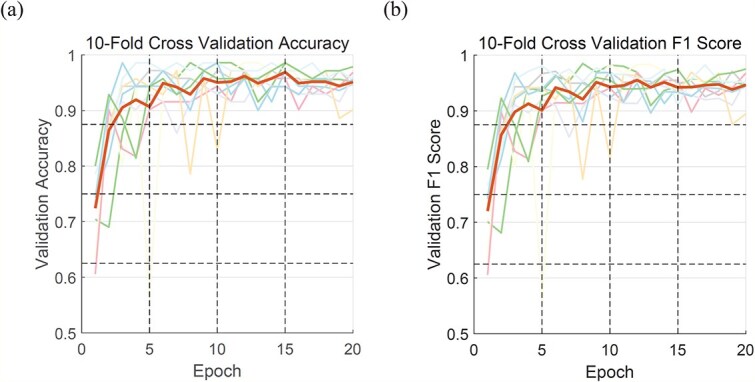
(a) Ten-fold cross validation ACC convergence plot. (b) Ten-fold cross validation f1-score convergence plot.

We used the datasets constructed above to predict the phase separation characteristics of proteins under various conditions. We implemented 10-fold cross-validation during training to enhance model robustness [36], as illustrated in Fig. 3. The experimental results demonstrate that our model exhibits excellent performance in predicting protein phase separation, achieving an accuracy of 95.2% and an F1-score [37] of 96.4%, surpassing existing models. This result indicates that our model possesses outstanding reliability and application potential in identifying and distinguishing LLPS proteins, providing a solid foundation for further research. As shown in Table 1, we compared the performance of MambaPhase with several state-of-the-art methods including PSPredictor [38], PScore [39], PLAAC, and catGRA, on the SaPS_test dataset. Using the aforementioned assessment metrics, our approach clearly demonstrated superior performance.

**Table 1:** Comparison of model performance metrics

**Method**	**Accuracy**	**F1-Score**	**Precision**	**MCC**
PScore	0.7622	0.3598	**1**	0.8629
PLAAC	0.8266	0.4641	**1**	0.8752
catGRA	0.5578	0.4515	0.3254	0.8579
PSPredictor	0.9471	0.9466	0.9612	0.8970
PhaSePred	0.8456	0.6981	**1**	0.8591
PredLLPS_PSSM	0.9328	0.8648	0.8718	0.8861
**MambaPhase**	**0.9521**	**0.9642**	0.9269	**0.8981**

### Distinction evaluation of scaffold proteins, client proteins, and pH and salt concentrations

After identifying potential phase-separating proteins (PSPs), we conducted a more detailed classification to deeply analyze the characteristic differences between client proteins and scaffold proteins. The training and test sets were sourced from the seq2phase paper [40]. As demonstrated in Table 2, our method was compared with the leading existing models, and the results demonstrate that our model exhibits superior performance.

**Table 2:** ROC-AUC for client protein classification

**Method**	**ROCAUC Score**
**MambaPhase**	**0.872**
Seq2Phase	0.859
425 features	0.781

We performed comparative experiments on pH values and salt concentrations using datasets previously deduplicated by the CD-Hit algorithm. The datasets were split into training and test sets in an 8:2 ratio. We compared our approach with traditional methods based on physicochemical properties and machine learning [41]. The results showed in Table 3 demonstrate that our network model achieved optimal performance.

**Table 3:** Compared with traditional machine learning methods, contrastive learning demonstrates superior performance in classifying experimental conditions such as salt concentration and pH

**Algorithm**	**Ph**	**Salt**
Logistic Regression	0.70	0.66
K-Nearest Neighbors	0.68	0.69
Support Vector Machine (linear)	0.66	0.68
Decision Tree	0.63	0.58
Random Forest	0.73	0.68
Gradient Boosting	0.70	0.68
MLP Classifier	0.70	0.68
**MambaPhase**	**0.77**	**0.69**

To validate the generalizability of our model to PSPs not included in the training set, as well as to evaluate its predictive capability for drug delivery systems, we selected the HBpep PSP [42], **which had been experimentally validated and published in Nature, for further assessment. Using the CD-Hit clustering algorithm with an identity threshold of 40%,** we confirmed that this sequence was absent from our training dataset. PSPs such as HBpep have attracted increasing attention for their capability to form responsive drug delivery systems due to their tunable physicochemical properties and controlled release behaviors. These characteristics allow them to entrap therapeutic agents effectively and release them in a controlled manner upon environmental stimuli such as pH, temperature, or ionic strength fluctuations. Such environmental responsiveness makes these proteins highly attractive for biomedical applications, including smart drug delivery systems [43].

Indeed, similar phase separating biomaterials have been employed successfully elsewhere as stimulus-responsive insulin release systems. For example, in the insulin delivery system named DgHBP-2 [44], the peptide GHGVYGHGVYGHGPY GHGPYGHGLYW (DgHBP-2), comprising 26 amino acids derived from a histidine-rich beak protein originally identified in the Humboldt squid (Dosidicus gigas), has significant biomedical applications, particularly in insulin delivery systems design. This peptide can readily undergo phase separation under neutral to slightly alkaline conditions, forming coacervate microdroplets capable of encapsulating therapeutic proteins such as insulin and glucose oxidase with very high efficiency. Upon exposure to glucose, glucose oxidase rapidly converts glucose to gluconic acid, lowering the local pH, thereby triggering the release of encapsulated insulin. This peptide’s structure facilitates the development of insulin delivery vehicles capable of responding dynamically and reversibly to fluctuations in glucose levels, mimicking the physiological functions of pancreatic $\beta $-cells. Such glucose-responsive insulin delivery systems can automatically release insulin according to blood glucose levels, which is crucial for effectively managing diabetes. The utilization of such phase-separated peptides enables efficient and precise insulin delivery, potentially reducing the need for repeated insulin injections and significantly improving patients’ quality of life by decreasing their direct involvement in glucose control.

**Figure 4:**
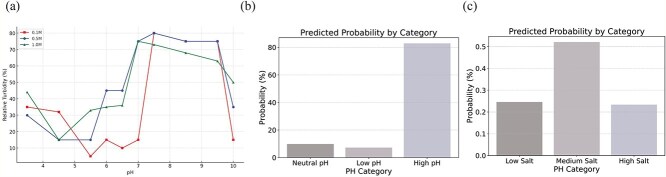
(a) A plot of turbidity changes in the DgHBP-2 paper, where it can be seen that turbidity increases at pH above 7 and salt concentrations between 0.1M and 0.5M. (b) Range of phase separation pH given by the model. (c) Range of salt concentration for phase separation given by the model.

Figure 4, taken from the DgHBP-2 study, illustrates how variations in turbidity correlate directly with phase separation events, where a marked increase in turbidity signals the onset of coacervate formation. The experimental results demonstrate clearly that prominent phase separation occurs within a pH range of $\sim $7–9, indicating that mildly alkaline environments are favorable for initiating the phase separation process. Additionally, the degree of phase separation, as reflected by changes in turbidity, exhibits a noticeable dependence on salt concentration. More specifically, turbidity—and thus phase separation—is significantly enhanced at intermediate salt concentrations, particularly in a range from 0.1M to 0.5M, suggesting an optimal ionic environment to stabilize coacervate droplets and facilitate robust phase transitions.

When the DgHBP-2 sequence was input into our predictive model, the model successfully identified the peptide sequence as a scaffold protein and accurately predicted its capability to undergo phase separation at pH values exceeding 7, consistent with experimental findings. Furthermore, our modeling forecasted that effective phase separation would be most likely observed within a narrower salt concentration window, specifically between 0.1M and 0.3M, closely aligning with the key region observed experimentally. Although experimental results suggest that a broader salt concentration range (0.1M to 0.5M) could facilitate phase separation due to enhanced turbidity measurements at its extremes (0.1M and 0.5M), it is noteworthy that minimal phase separation activity—indicated by low turbidity—was detected at higher salt levels, particularly at 1.0M concentration. Consistent with this observation, our model predicts a decrease in the probability of phase separation at concentrations above $\sim $0.3M, with a secondary yet relatively lower probability predicted for the interval spanning 0.3M to 0.5M. Thus, although minor variance exists between our model’s predictions and specific experimental turbidity data at higher ionic strengths (around 0.5M), the overall good alignment suggests that the model reliably identifies key threshold conditions necessary for phase separation. Consequently, such predictive capability provides researchers with a valuable preliminary basis to narrow down the experimental conditions to be tested—accelerating discovery, streamlining experimental resources, and facilitating the rational design of peptide-based phase-separated systems for biomedical and therapeutic applications.

## Conclusion

The study efficiently identified key proteins involved in the LLPS process, including scaffold and client proteins, through the development of the MambaPhase model. Experimental results indicated that the model achieved a 95.2% accuracy in predicting phase separation probability and an ROCAUC of 0.87 in differentiating between scaffold and client proteins, demonstrating strong classification capabilities. Additionally, the model achieved an overall ROCAUC of 77% in separately predicting conditions involving pH values and salt concentrations, underscoring MambaPhase’s strong predictive performance in recognizing suitable experimental conditions for phase separation.

Unlike traditional LLPS prediction methods, our approach integrates the protein language model ESM and employs contrastive learning combined with the Mamba network. This integration allows the MambaPhase model to discern subtle differences among protein sequences even with limited data, thereby significantly reducing dependence on large datasets and exhibiting robust generalization capabilities. We further applied the MambaPhase model to predict the phase separation behavior in the drug delivery system DgHBP-2, validating the model’s predictive sensitivity regarding changes in pH values and salt concentrations. These findings highlight the MambaPhase model’s exceptional potential for guiding and informing precise experimental condition selection during the optimization of drug delivery systems.

Future research will aim to expand the model’s application to investigate phase separation behavior across additional diverse experimental conditions, focusing particularly on challenging classification tasks involving broader ranges of proteins and their interactions. Furthermore, current categorizations of salt concentrations and pH values are not exhaustive, and exploring alternative classification strategies remains an important avenue for further refinement. Continued acquisition and incorporation of additional data will allow us to provide more nuanced, accurate support for LLPS predictions, thus facilitating advancements in both fundamental LLPS studies and biomedical applications in drug delivery systems.

**Key Points**

- The model uses ESM and Mamba, reducing data dependency and demonstrating robust generalization, validated in drug delivery systems.
- MambaPhase achieved 95.2% accuracy in predicting LLPS and strong classification for scaffold versus client proteins and experimental conditions.
- Initially, we examined the experimental conditions for LLPS and visualized the differences in the physicochemical properties of proteins undergoing phase separation under varying conditions. Additionally, we developed a model for LLPS capable of classifying into three distinct levels based on pH and salt concentration.

## Data Availability

The models and datasets used in this article are available at https://github.com/biabiubong/Mambaphase
